# Health Care Affordability Problems by Income Level and Subsidy Eligibility in Medicare

**DOI:** 10.1001/jamanetworkopen.2025.32862

**Published:** 2025-09-22

**Authors:** Sungchul Park, Vicki Fung

**Affiliations:** 1 Department of Health Policy and Management, College of Health Science, BK21 FOUR R&E Center for Learning Health Systems, Korea University, Seoul, Republic of Korea; 2 Mongan Institute Health Policy Research Center, Massachusetts General Hospital, Harvard Medical School, Boston

## Abstract

**Importance:**

Standard Medicare benefits include substantial cost sharing, often resulting in high out-of-pocket expenses for beneficiaries. Financial assistance programs help mitigate these costs by covering Medicare Parts A and B premiums and cost sharing for beneficiaries with limited assets and incomes below 100% of the federal poverty level (FPL) and premiums for beneficiaries with incomes between 100% and 150% of the FPL.

**Objective:**

To examine health care affordability problems among Medicare beneficiaries by income levels aligned with subsidy eligibility.

**Design, Setting, and Participants:**

This cross-sectional study of Medicare beneficiaries using data from the 2018-2022 Medical Expenditure Panel Survey was conducted from December 2024 through March 2025.

**Exposure:**

Household income categorized into 4 groups: low income (<100% of FPL), near low income (100%-150% of FPL), middle income (150%-400% of FPL), and high income (>400% of FPL).

**Main Outcomes and Measures:**

Three indicators of health care affordability problems: financial burden (spending ≥20% of annual postsubsistence income on out-of-pocket costs), financial barriers to care (delaying or forgoing care due to cost), and medical debt.

**Results:**

The sample included 24 398 Medicare beneficiaries (mean [SD] age, 71.6 [9.3] years; 54.9% female). Out-of-pocket spending increased with increasing income: $1363 (95% CI, $1301-$1425) for beneficiaries with near low income, $1765 (95% CI, $1571-$1958) for beneficiaries with low income, $2611 (95% CI, $2512-$2710) for beneficiaries with middle income, and $3964 (95% CI, $3875-$4054) for beneficiaries with high income. However, beneficiaries with near low income faced the highest levels of health care affordability challenges compared with the other income groups. After adjusting for age, sex, and health status, 54.0% (95% CI, 52.7%-55.3%) of beneficiaries with near low income reported experiencing at least 1 affordability problem, compared with 43.0% (95% CI, 40.5%-45.5%) in the low-income group, 45.2% (95% CI, 44.1%-46.4%) in the middle-income group, and 25.5% (95% CI, 24.7%-26.4%) in the high-income group. These patterns remained consistent across all affordability measures. Although few beneficiaries experienced all 3 types of affordability problems, larger proportions of beneficiaries reported financial burden alone, financial barriers to care alone, or both without medical debt.

**Conclusions and Relevance:**

In this cross-sectional study of Medicare beneficiaries, health care affordability problems were common. Medicare beneficiaries with near low income, who qualified for only partial financial assistance with Medicare costs, faced greater financial hardship compared with beneficiaries in other income groups. These affordability challenges may have restricted access to needed care, highlighting the need for targeted strategies to enhance financial protections and improve health care affordability for these beneficiaries.

## Key Points

**Question:**

How do health care affordability problems vary by income level and subsidy eligibility among Medicare beneficiaries?

**Findings:**

In this cross-sectional study of 24 398 Medicare beneficiaries, beneficiaries with lower income had lower out-of-pocket health care spending than Medicare beneficiaries with higher income, but they were more likely to experience affordability challenges. Beneficiaries with near low income (100%-150% of the federal poverty level [FPL]) reported the highest levels of affordability problems—54.0%—exceeding those in the low-income (<100% of FPL; 43.0%), middle-income (150%-400% of FPL; 45.2%), and high-income (>400% of FPL; 25.5%) groups.

**Meaning:**

This study suggests that Medicare beneficiaries with near low income are particularly likely to experience affordability challenges, likely due to limited financial support.

## Teaser

This cross-sectional study uses data from the 2018-2022 Medical Expenditure Panel Survey to examine health care affordability problems among Medicare beneficiaries by income levels aligned with subsidy eligibility.

## Introduction

Although Medicare provides extensive coverage for essential health care services, standard Medicare benefits include substantial cost sharing, which can lead to high out-of-pocket (OOP) expenses for beneficiaries.^1,2^ Traditional Medicare requires beneficiaries to pay deductibles and coinsurance for inpatient and outpatient care, with no cap on OOP spending.^3^ Although Medicare Advantage plans impose OOP spending limits, these thresholds can exceed $9000.^4^ In 2022, Medicare households spent an average of $7000 on health care, with about one-third of beneficiaries overall spending 20% or more of their total income on medical expenses.^5^ This burden is particularly concerning for Medicare beneficiaries with low income, as nearly half of those with incomes below 150% of the federal poverty level (FPL) spent at least 20% of their income on health care costs.^6^

To mitigate these financial challenges, income-based subsidies are available through the Medicare Savings Program and the Part D Low-Income Subsidy (LIS) program, both of which determine eligibility using income and asset tests. Beneficiaries with incomes at or below 100% of the FPL and limited assets in most states qualify for Medicaid assistance with Medicare Parts A and B premiums and cost sharing through the Qualified Medicare Beneficiary program. These beneficiaries also automatically qualify for the full Part D LIS program, which reduces premiums and cost sharing for prescription drugs. Beneficiaries with incomes between 100% and 150% of the FPL are eligible for assistance with Part B premiums (but not cost sharing) through the Specified Low-Income Medicare Beneficiary and Qualified Individual programs, as well as the full or partial Part D LIS program. Those with incomes above 150% of the FPL do not quality for any of these subsidies. Income tests generally include earned and unearned income but exclude a portion of earned income; asset tests include money in bank accounts and investments but exclude certain assets, such as a beneficiary’s primary home.

To inform effective policies that improve access to care for Medicare beneficiaries, it is essential to understand how health care affordability varies across income levels as well as the availability of public assistance.^7,8^ Qualified Medicare Beneficiary subsidies are associated with lower OOP spending and higher health care use among beneficiaries with lower income.^9,10,11,12^ However, beneficiaries with near low income, defined as those with incomes between 100% and 150% of the FPL, who are generally not eligible for Medicare cost-sharing subsidies, often face similar social determinants of health as beneficiaries with low income and experience significant affordability concerns.^6,13,14^ Beneficiaries with incomes just above the threshold for the Part D LIS program reported lower medication adherence and greater financial stress due to drug costs compared with those in other income groups, including beneficiaries with incomes below the FPL.^15^ Furthermore, the use of high-value care is lower among older adults with lower income.^16^ These findings raise concerns about affordability of care and questions about whether the current sources of financial assistance adequately protect beneficiaries with near low income from financial hardship.

To address these knowledge gaps, we examined health care affordability problems among Medicare beneficiaries by income levels that align with subsidy eligiblity. Specifically, we conducted 3 analyses. First, we assessed health care spending and utilization patterns across income levels. Second, we evaluated 3 indicators of health care affordability problems (financial burden, financial barriers to care, and medical debt) across income levels. Third, recognizing that these indicators capture distinct dimensions of health care financial hardship,^17^ we examined their joint distribution to provide a comprehensive understanding of the financial difficulties faced by beneficiaries.

## Methods

### Data Source

We conducted a cross-sectional study using data from the 2018-2022 Medical Expenditure Panel Survey (MEPS), a nationally representative survey of the US noninstitutionalized population.^18^ The 2018-2022 period was chosen because complete data for all 3 indicators of health care affordability problems were available during these years. The MEPS is reviewed and approved annually by the Westat institutional review board. Our study used data that are publicly available and deidentified, and was therefore exempt from institutional review board approval and informed consent requirements, in accordance with the Common Rule. We adhered to the Strengthening the Reporting of Observational Studies in Epidemiology (STROBE) reporting guideline for observational studies.

### Sample

We initially identified 31 289 Medicare beneficiaries and excluded 6891 beneficiaries with missing data, resulting in a final sample of 24 398 Medicare beneficiaries with complete information. The sample selection process is illustrated in eFigure 1 in Supplement 1; the final sample was similar to the total sample in observable characteristics.

### Outcomes

Our primary outcomes were health care spending, health care use, and affordability problems. Health care spending was measured through both total and OOP expenditures. All spending measures were adjusted to 2021 US dollars using the Personal Consumption Expenditures Price Index^19^ for health care. Health care use was assessed by the numbers of inpatient visits, outpatient visits, emergency department visits, and prescription drug fills per 100 persons per year. Health care affordability problems were evaluated using 3 binary indicators (financial burden, financial barriers to care, and medical debt), which were developed based on prior research.^17^ The first indicator, financial burden, was defined as families spending 20% or more of their annual postsubsistence income on OOP costs, including premiums and medical care. Postsubsistence income was calculated by deducting food-related expenses, based on food cost nomograms provided by the US Bureau of Labor Statistics.^20^ The second indicator, financial barriers to care, was derived from MEPS questions asking whether any household member experienced delays in treatment or went without medical, dental, or prescription medication treatment due to costs. The third indicator, medical debt, was assessed using MEPS questions asking whether any family member had outstanding medical bills, including those paid with credit cards or personal loans, that were being paid over time or remained unpaid. To avoid misclassifying installment payments for bundled services, such as orthodontic care, as medical debt, bills paid over time were only classified as debt if reported as a financial problem. In addition to these specific affordability measures, we constructed binary indicators to assess whether respondents experienced any of the 3 types of affordability problems. Furthermore, we developed 7 mutually exclusive binary indicators to assess the joint distribution of these indicators: financial burden only, financial barrier only, medical debt only, financial burden and financial barrier, financial burden and medical debt, medical debt and financial barrier, and a combination of all 3.

### Primary Independent Variable

The key independent variable was household income as a percentage of the FPL, categorized into 4 groups based on subsidy eligibility: low income (<100% of FPL), near low income (100%-150% of FPL), middle income (150%-400% of FPL), and high income (>400% of FPL). Income was measured at the household level by summing all income sources from all earners within the household. The total household income was then divided by the poverty threshold, adjusted for family size and composition, for each analysis year. MEPS provides an inflation-adjusted FPL to ensure comparability across survey years.

### Covariates

To examine sample characteristics by income group, we compared demographic, health, and socioeconomic factors. Demographic factors included age and sex. Health factors included the number of chronic conditions, self-reported physical and mental health, and Physical and Mental Component Summary scores from the 12-item Short Form Health Survey. Socioeconomic factors included self-reported race and ethnicity (Hispanic, non-Hispanic Asian, non-Hispanic Black, non-Hispanic White, and non-Hispanic other or multiple races [non-Hispanic individuals who identify as American Indian or Alaska Native, Native Hawaiian or Other Pacific Islander, or those who report ≥2 races]), employment status, marital status, educational level, household income, health insurance, and US Census region of residence. Data on race and ethnicity were collected because health care utilization differs by race and ethnicity, and we believe adjusting for this factor is necessary to enable more accurate comparisons.

### Statistical Analysis

The analysis was conducted from December 2024 through March 2025. We compared sample characteristics by income group. Next, we calculated the mean unadjusted outcome values for each group. To estimate adjusted outcomes, we used a 2-part model for health care spending, a linear regression model for health care use, and a logistic regression model for health care affordability problems. According to the Institute of Medicine, disparities are defined as differences in the quality of health care that are not due to differences in patients’ health care needs or preferences.^21,22^ In line with the Institute of Medicine framework, we followed prior research by adjusting only for demographic factors (age and sex) and health factors to distinguish differences related to need from differences related to other systemic factors that could be associated with inequities.^22^ Using marginal effects from these models, we estimated the mean adjusted values of the outcomes for each group while holding all other variables constant, except the variable of interest. We also estimated the adjusted differences in the outcomes between each income group and the reference group (beneficiaries with near low income).

For all analyses, we clustered standard errors at the individual level. We applied survey weights to generate nationally representative estimates and to account for the complex survey design in variance estimation. All statistical analyses were conducted using Stata, version 17.0 (StataCorp LLC).

## Results

### Sample Characteristics

Our sample consisted of 24 398 Medicare beneficiaries (mean [SD] age, 71.6 [9.3] years; 54.9% women and 45.1% men; 18.1% Hispanic beneficiaries, 5.8% non-Hispanic Asian beneficiaries, 12.2% non-Hispanic Black beneficiaries, 60.0% non-Hispanic White beneficiaries, and 3.6% beneficiaries of other race and ethnicity) (Table 1). The sample included 3811 beneficiaries with low income, 2894 beneficiaries with near low income, 9115 beneficiaries with middle income, and 8578 beneficiaries with high income. Sample characteristics varied by income group. Compared with beneficiaries with middle income or with high income, beneficiaries with low income or with near low income reported worse health, were more likely to be members of racial and ethnic minority groups, had lower employment rates, were more likely to report having Medicaid coverage, and were less likely to report having private insurance coverage. However, differences between beneficiaries with low income and beneficiaries with near low income were relatively small, particularly with regard to differences in health factors. Among beneficiaries with low income vs beneficiaries with near low income, the prevalence of 3 to 5 chronic conditions was 17.7% vs 17.1% compared with 13.1% of beneficiaries with middle income and 9.9% of beneficiaries with high income. In addition, 62.8% of beneficiaries with low income and 67.3% of beneficiaries with near low income reported excellent, very good, or good physical health compared with 77.8% of beneficiaries with middle income and 87.6% of beneficiaries with high income.

**Table 1.** Characteristics of Medicare Beneficiaries by Income Level and Subsidy Eligibility

Characteristic	Medicare beneficiaries, weighted %^a^			
	Low income (100% of FPL) (n = 3811)	Near low income (101%-150% of FPL) (n = 2894)	Middle income (151%-399% of FPL) (n = 9115)	High income (≥400% of FPL) (n = 8578)
Demographic factors				
Age, y				
<65	31.0	19.7	10.2	4.7
65-74	37.9	38.1	49.5	61.2
75-84	21.6	27.4	29.3	27.8
≥85	9.6	14.8	11.0	6.2
Sex				
Male	40.2	39.5	43.5	49.2
Female	59.8	60.5	56.5	50.8
Health factors				
No. of chronic conditions				
0	30.4	32.8	38.5	43.6
1-2	50.6	49.0	47.7	46.2
3-5	17.7	17.1	13.1	9.9
≥6	1.3	1.1	0.7	0.2
Self-reported good health				
Physical	62.8	67.3	77.8	87.6
Mental	75.9	80.4	86.6	93.2
Health-related quality of life, mean (SD)				
SF-12 physical component score	37.5 (12.4)	37.9 (12.3)	41.2 (12.2)	45.7 (11.3)
SF-12 mental component score	48.1 (11.4)	48.9 (11.1)	51.8 (9.5)	53.9 (7.9)
Socioeconomic factors				
Race and ethnicity				
Hispanic	14.0	11.5	8.3	3.8
Non-Hispanic Asian	4.4	2.6	2.9	3.9
Non-Hispanic Black	20.0	14.6	10.3	5.3
Non-Hispanic White	57.5	68.0	76.0	85.6
Non-Hispanic other or multiple races^b^	4.0	3.3	2.5	1.4
Employed	4.2	6.9	15.9	31.0
Married	29.4	26.1	47.4	70.8
Educational level				
No high school diploma	23.7	20.5	11.1	2.6
High school graduate	53.0	56.7	54.8	35.9
College graduate or higher	22.5	22.1	33.7	61.2
Health insurance coverage				
Medicare benefits				
Traditional Medicare	58.7	53.3	55.1	64.2
Medicare Advantage	41.3	46.7	44.9	35.8
Medicaid benefits				
Medicaid	44.1	24.9	9.5	2.3
No Medicaid	55.9	75.1	90.5	97.7
Other supplemental coverage				
Employer, Medigap, other	17.3	20.3	37.6	58.5
No other supplemental coverage	82.7	79.7	62.4	41.5
US Census region				
Northeast	14.7	16.6	17.0	18.2
Midwest	19.6	22.3	23.1	23.7
South	47.3	43.3	39.3	34.1
West	18.4	17.9	20.6	23.9

Abbreviations: FPL, federal poverty level; SF-12, 12-item Short Form Health Survey.

^a^ Survey weights were applied to ensure the sample was representative of the Medicare population and to account for the complex survey design in standard error estimation.

^b^ Includes non-Hispanic individuals who identify as American Indian or Alaska Native, Native Hawaiian or Other Pacific Islander, or those who report 2 or more races.

### Health Care Spending and Use

Health care spending varied substantially by income group (Table 2). Unadjusted total health care spending decreased and OOP spending increased from the lowest to highest income category. However, after adjusting for demographic and health factors, total spending increased with income, suggesting that beneficiaries with lower income had lower health care spending conditional on clinical need. The adjusted mean total health care spending was $11 890 (95% CI, $11 479-$12 302) for beneficiaries with low income, $12 831 (95% CI, $12 419-$13 243) for beneficiaries with near low income, $13 369 (95% CI, $13 006-$13 732) for beneficiaries with middle income, and $15 015 (95% CI, $14 442-$15 588) for beneficiaries with high income. Similarly, the adjusted mean OOP spending was $1363 (95% CI, $1301-$1425) for beneficiaries with low income, $1765 (95% CI, $1571-$1958) for beneficiaries with near low income, $2611 (95% CI, $2512-$2710) for beneficiaries with middle income, and $3964 (95% CI, $3875-$4054) for beneficiaries with high income.

**Table 2.** Health Care Spending and Use Among Medicare Beneficiaries by Income Level and Subsidy Eligibility

Outcome	Income level and subsidy eligibility^a^			
	Low income (100% of FPL)	Near low income (101%-150% of FPL)	Middle income (151%-399% of FPL)	High income (≥400% of FPL)
**Health care spending**				
Total spending, $				
Unadjusted mean (SD)	18 563 (34 143)	18 223 (30 277)	15 972 (33 169)	14 427 (26 414)
Adjusted mean (95% CI)	11 890 (11 479 to 12 302)	12 831 (12 419 to 13 243)	13 369 (13 006 to 13 732)	15 015 (14 442 to 15 588)
Adjusted difference (95% CI)	−941 (−1498 to −383)	1 [Reference]	538 (−83 to 1159)	2184 (1444 to 2924)
OOP spending, $				
Unadjusted mean (SD)	1352 (2984)	1814 (6223)	2660 (5543)	3877 (6350)
Adjusted mean (95% CI)	1363 (1301 to 1425)	1765 (1571 to 1958)	2611 (2512 to 2710)	3964 (3875 to 4054)
Adjusted difference (95% CI)	−402 (−587 to −216)	1 [Reference]	846 (721 to 972)	2200 (1991 to 2409)
**Health care use**				
No. of inpatient admissions per 100 persons				
Unadjusted mean (SD) No. of visits	25.0 (63.9)	28.6 (69.9)	21.3 (61.5)	15.6 (50.5)
Adjusted mean No. of visits (95% CI)	18.8 (17.1 to 20.5)	22.7 (20.0 to 25.4)	20.1 (18.5 to 21.7)	19.8 (18.4 to 21.3)
Adjusted difference in No. of visits (95% CI)	−3.9 (−5.6 to −2.3)	1 [Reference]	−2.6 (−4.2 to −1.1)	−2.9 (−6.8 to 1.1)
No. of clinician visits per 100 persons				
Unadjusted mean (SD) No. of visits	2233.6 (3007.5)	2238.9 (2713.1)	2286.8 (2578.3)	2379.4 (2492.0)
Adjusted mean No. of visits (95% CI)	1866.6 (1734.8 to 1998.4)	1962.6 (1918.3 to 2006.9)	2245.1 (2112 to 2378.1)	2586.1 (2533.8 to 2638.4)
Adjusted difference in No. of visits (95% CI)	−96.0 (−260.4 to 68.4)	1 [Reference]	282.5 (120.2 to 444.8)	623.5 (565.4 to 681.7)
No. of ED visits per 100 persons				
Unadjusted mean (SD) No. of visits	46.5 (99.3)	45.8 (90.6)	36.6 (83.9)	25.9 (71.1)
Adjusted mean No. of visits (95% CI)	35.2 (33.5 to 36.9)	36.4 (32.9 to 39.9)	35.1 (33.9 to 36.4)	32.7 (29.3 to 36.2)
Adjusted difference in No. of visits (95% CI)	−1.2 (−5.8 to 3.3)	1 [Reference]	−1.2 (−3.8 to 1.3)	−3.7 (−9.8 to 2.5)
No. of prescription drug fills per 100 persons				
Unadjusted mean (SD) No. of visits	3462.1 (3702.6)	3198.0 (3338.4)	2569.5 (2739.0)	2045.5 (2069.5)
Adjusted mean No. of visits (95% CI)	2776.7 (2598.2 to 2955.1)	2711.1 (2604.9 to 2817.3)	2514.8 (2468.3 to 2561.3)	2403.2 (2345.0 to 2461.4)
Adjusted difference in No. of visits (95% CI)	65.5 (−178.4 to 309.5)	1 [Reference]	−196.3 (−304.9 to −87.8)	−307.9 (−371.8 to −244.0)

Abbreviations: ED, emergency department; FPL. federal poverty level; OOP, out-of-pocket.

^a^ Regression analyses were performed using a 2-part model for health care spending and a linear regression model for health care use. Adjusted mean values and differences were derived from the regression models after adjusting for demographic (age and sex) and health characteristics (number of chronic conditions, self-reported physical and mental health, and Physical and Mental Component Summary scores from the 12-item Short Form Health Survey) as well as year fixed effects. Survey weights were applied to ensure the sample was representative of the Medicare population and to account for the complex survey design in standard error estimation.

Patterns of health care use varied by type of care (Table 2). Beneficiaries with middle income or with high income had more clinician visits compared with beneficiaries with near low income after adjusting for demographics and health factors, but they had lower prescription drug use. There were no significant differences in clinician visits and prescription drug use between beneficiaries with low income and beneficiaries with near low income. Inpatient admissions and emergency department visits were most frequent among beneficiaries with near low income. The adjusted number of inpatient admissions per 100 persons was 22.7 (95% CI, 20.0-25.4) among beneficiaries with near low income compared with 18.8 (95% CI, 17.1-20.5) in the low-income group, 20.1 (95% CI, 18.5-21.7) in the middle-income group, and 19.8 (95% CI, 18.4-21.3) in the high-income group. Similarly, the adjusted number of emergency department visits per 100 persons was 36.4 (95% CI, 32.9-39.9) for beneficiaries with near low income compared with 35.2 (95% CI, 33.5-36.9) in the low-income group, 35.1 (95% CI, 33.9-36.4) in the middle-income group, and 32.7 (95% CI, 29.3-36.2) in the high-income group. For these measures, there were no significant differences across income levels.

### Health Care Affordability Problems

Beneficiaries with near low income reported the highest levels of health care affordability problems across all 3 indicators in both unadjusted and adjusted analyses (Table 3). After adjustment, 54.0% (95% CI, 52.7-55.3) of beneficiaries with near low income reported at least 1 health care affordability problem compared with 43.0% (95% CI, 40.5%-45.5%) of beneficiaries with low income, 45.2% (95% CI, 44.1%-46.4%) of beneficiaries with middle income, and 25.5% (95% CI, 24.7%-26.4%) of beneficiaries with high income. The adjusted likelihood of reporting any affordability problem was lower by 11.0 percentage points (95% CI, −13.5 to −8.6 percentage points), 8.8 percentage points (95% CI, −11.1 to −6.5 percentage points), and 28.5 (95% CI, −29.9, −27.0 percentage points), respectively.

**Table 3.** Health Care Affordability Problems Among Medicare Beneficiaries by Income Level and Subsidy Eligibility

Outcome	Income level and subsidy eligibility^a^			
	Low income (100% of FPL)	Near low income (101%-150% of FPL)	Middle income (151%-399% of FPL)	High income (≥400% of FPL)
**Any affordability problem**				
Unadjusted mean (SD) %	46.5 (5.0)	56.1 (5.0)	45.4 (5.0)	24.1 (4.3)
Adjusted mean % (95% CI)	43.0 (40.5 to 45.5)	54.0 (52.7 to 55.3)	45.2 (44.1 to 46.4)	25.5 (24.7 to 26.4)
Adjusted difference, percentage points (95% CI)	−11.0 (−13.5 to −8.6)	1 [Reference]	−8.8 (−11.1 to −6.5)	−28.5 (−29.9 to −27.0)
**Financial burden**				
Unadjusted mean (SD) %	27.1 (4.4)	33.6 (4.7)	28.0 (4.5)	13.9 (3.5)
Adjusted mean % (95% CI)	28.6 (26.8 to 30.4)	34.1 (33.4 to 34.7)	27.7 (26.3 to 29.1)	13.6 (13.3 to 13.9)
Adjusted difference, percentage points (95% CI)	−5.5 (−7.7 to −3.2)	1 [Reference]	−6.4 (−8.3 to −4.4)	−20.5 (−21.3 to −19.7)
**Medical debt**				
Unadjusted mean (SD) %	6.5 (2.5)	9.3 (2.9)	5.1 (2.2)	2.0 (1.4)
Adjusted mean % (95% CI)	4.0 (3.1 to 4.8)	7.1 (6.1 to 8.1)	5.2 (4.8 to 5.5)	2.7 (2.3 to 3.1)
Adjusted difference, percentage points (95% CI)	−3.1 (−4.5 to −1.7)	1 [Reference]	−1.9 (−2.8 to −1.0)	−4.4 (−5.5 to −3.2)
**Financial barrier**				
Unadjusted mean (SD) %	25.2 (4.3)	30.4 (4.6)	21.0 (4.1)	11.0 (3.1)
Adjusted mean % (95% CI)	19.9 (18.0 to 21.8)	26.9 (26.3 to 27.6)	20.9 (20.0 to 21.8)	12.6 (12.2 to 13.0)
Adjusted difference, percentage points (95% CI)	−7.0 (−8.6 to −5.5)	1 [Reference]	−6.0 (−6.4 to −5.6)	−14.3 (−15.0 to −13.6)

Abbreviation: FPL, federal poverty level.

^a^ Following prior research, we constructed 3 indicators of health care financial problems (financial burden, financial barriers to care, and medical debt). Using these indicators, we created binary measures to assess whether beneficiaries experienced any of the 3 financial problems and conducted further analyses to examine each problem separately. For each indicator, regression analyses were conducted using a logistic regression model. Adjusted mean values and differences were derived from the regression models after adjusting for demographic (age and sex) and health characteristics (number of chronic conditions, self-reported physical and mental health, and Physical and Mental Component Summary scores from the 12-item Short Form Health Survey) as well as year fixed effects. Survey weights were applied to ensure the sample was representative of the Medicare population and to account for the complex survey design in standard error estimation.

For specific indicators, 34.1% (95% CI, 33.4%-34.7%) of beneficiaries with near low income reported experiencing financial burden compared with 28.6% (95% CI, 26.8%-30.4%) of beneficiaries with low income, 27.7% (95% CI, 26.3%-29.1%) of beneficiaries with middle income, and 13.6% (95% CI, 13.3%-13.9%) of beneficiaries with high income (Table 3). In addition, 26.9% (95% CI, 26.3%-27.6%) of beneficiaries with near low income reported facing financial barriers to care compared with 19.9% (95% CI, 18.0%-21.8%) of beneficiaries with low income, 20.9% (95% CI, 20.0%-21.8%) of beneficiaries with middle income, and 12.6% (95% CI, 12.2%-13.0%) of beneficiaries with high income. Finally, 7.1% (95% CI, 6.1%-8.1%) of beneficiaries with near low income reported medical debt compared with 4.0% (95% CI, 3.1%-4.8%) of beneficiaries with low income, 5.2% (95% CI, 4.8%-5.5%) of beneficiaries with middle income, and 2.7% (95% CI, 2.3%-3.1%) of beneficiaries with high income.

We adjusted for year fixed effects but also examined the trends directly. We found that health care use decreased after the onset of the COVID-19 pandemic, at the same time as a decrease in the prevalence of reported affordability problems over time (eFigure 2 in Supplement 1).

### Joint Distribution of Health Care Affordability Problems

The proportion of beneficiaries reporting each health care affordability problem in the joint distribution was highest among beneficiaries with near low income, although similar patterns were observed across all income groups (Figure). Only a small proportion of beneficiaries experienced all 3 affordability problems (1.3% for beneficiaries with low income, 2.1% for beneficiaries with near low income, 0.7% for beneficiaries with middle income, and 0.3% for beneficiaries with high income) (Table 4). However, larger proportions reported either financial burden alone (21.5% for beneficiaries with low income, 24.2% for beneficiaries with near low income, 22.2% for beneficiaries with middle income, and 11.4% for beneficiaries with high income) or financial barriers to care alone (12.9% for beneficiaries with low income, 15.7% for beneficiaries with near low income, 13.5% for beneficiaries with middle income, and 9.6% for beneficiaries with high income).

**Figure.**
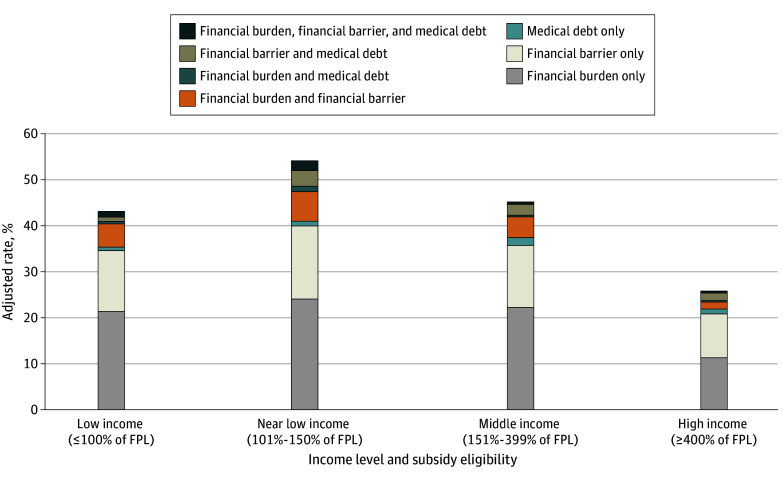
Joint Distribution of Health Care Affordability Problems Among Medicare Beneficiaries by Income Level and Subsidy Eligibility Using the 3 indicators of health care affordability problems (financial burden, financial barriers to care, and medical debt), we constructed 7 mutually exclusive binary measures for assessing their joint distribution: financial burden only, financial barrier only, medical debt only, financial burden and financial barrier, financial burden and medical debt, financial barrier and medical debt, and the combination of all 3 (financial burden, medical debt, and financial barrier). For each indicator, regression analyses were performed using a logistic regression model. Adjusted mean values and differences were estimated after controlling for demographic factors (age and sex), health characteristics (number of chronic conditions, self-reported physical and mental health, and Physical and Mental Component Summary scores from the 12-item Short Form Health Survey), and year fixed effects. Survey weights were applied to ensure the sample was representative of the Medicare population and to account for the complex survey design in standard error estimation. FPL indicates federal poverty level.

**Table 4.** Joint Distribution of Health Care Affordability Problems Among Medicare Beneficiaries by Income Level and Subsidy Eligibility

Outcome	Income level and subsidy eligibility, %^a^			
	Low income (100% of FPL)	Near low income (101%-150% of FPL)	Middle income (151%-399% of FPL)	High income (≥400% of FPL)
Financial burden only	21.5	24.2	22.2	11.4
Financial barrier only	12.9	15.7	13.5	9.6
Medical debt only	0.9	1.0	1.8	1.0
Financial burden and barrier	5.1	6.4	4.5	1.5
Financial burden and medical debt	0.6	1.3	0.4	0.2
Financial barrier and medical debt	0.9	3.3	2.2	1.7
Financial burden, barrier, and medical debt	1.3	2.1	0.7	0.3

Abbreviation: FPL, federal poverty level.

^a^ Using the 3 indicators of health care affordability problems (financial burden, financial barriers to care, and medical debt), we constructed 7 mutually exclusive binary measures for assessing their joint distribution: financial burden only, financial barrier only, medical debt only, financial burden and financial barrier, financial burden and medical debt, medical debt and financial barrier, and the combination of all 3 (financial burden, medical debt, and financial barrier). For each indicator, regression analyses were performed using a logistic regression model. Adjusted mean values and differences were estimated after controlling for demographic factors (age and sex), health characteristics (number of chronic conditions, self-reported physical and mental health, and Physical and Mental Component Summary scores from the 12-item Short Form Health Survey), and year fixed effects. Survey weights were applied to ensure the sample was representative of the Medicare population and to account for the complex survey design in standard error estimation.

## Discussion

We found that beneficiaries with low income and beneficiaries with near low income had lower OOP health care spending than beneficiaries with middle or high income. However, this spending pattern did not align with the experiences of health care affordability challenges. Although health care affordability problems were common across all income groups, beneficiaries with near low income faced the greatest financial hardship, with more than half of beneficiaries with near low income reporting at least 1 of the 3 types of financial hardship. These findings highlight the distinct economic challenges faced by individuals with near low income, who receive limited financial assistance yet continue to struggle with the cost of care.

Our study suggests significant income-related differences in health care spending and use. Consistent with prior studies,^16,23,24,25,26,27^ beneficiaries with lower income had poorer demographic, socioeconomic, and health characteristics than beneficiaries with higher income, which was associated with higher unadjusted health care spending. However, after adjusting for demographic and health factors, the spending pattern reversed, with beneficiaries with higher income incurring greater health care spending. This reversal suggests that beneficiaries with lower income have lower health care spending compared with beneficiaries with higher income with a similar health status. Although beneficiaries with lower income had lower OOP spending—likely due to financial assistance programs subsidizing premiums and/or cost sharing—this did not translate into greater use of outpatient care. Moreover, beneficiaries with low income and beneficiaries with near low income were more likely to use acute and emergency services, as reflected in higher inpatient admissions and emergency department visits. This pattern suggests that beneficiaries with lower income may face financial and logistical barriers that delay access to preventive and routine care, ultimately increasing the risk of more severe health conditions requiring urgent treatment.

Our study further highlights the distinct challenges faced by Medicare beneficiaries with near low income, who experience disproportionate financial strain. Beneficiaries with low income and beneficiaries with near low income had a similar health status, suggesting comparable underlying health needs. However, OOP health care expenses account for a disproportionately larger share of household income for beneficiaries with near low income compared with beneficiaries with low income. This financial burden is likely associated with the limited financial support available for individuals with near low income. Although beneficiaries with low income may receive more comprehensive financial protections through Medicaid and other assistance programs, individuals with near low income often fall into a critical coverage gap—earning too much to qualify for full assistance but still struggling to afford necessary care. Prior research has shown that the loss of comprehensive financial protections among beneficiaries with low income is associated with increased financial hardship and decreased use of health care services, often resulting in enrollment in lower-cost, potentially less-comprehensive health plans.^9,10,11,12^

Some recent federal and state policies aimed to improve health care affordability for Medicare beneficiaries with near low income by expanding eligibility, increasing generosity, and streamlining enrollment in assistance programs. In 2023, the Centers for Medicare & Medicaid Services finalized a rule to simplify enrollment in the Medicare Savings Program by aligning eligibility criteria and data systems with the Part D LIS program. Enhancing auto-enrollment in the Medicare Savings Program may particularly benefit beneficiaries with near low income eligible for Part B premium assistance through the Specified Low-Income Medicare Beneficiary or Qualified Individual programs, which historically have low participation rates—only about one-third of eligible individuals enrolled in the Specified Low-Income Medicare Beneficiary program in 2008 and 2009.^28^ However, the Budget Reconciliation Act passed in July 2025 delayed the implementation of many of these rules until 2035.^29^ In addition, 5 states and the District of Columbia have expanded income eligibility for the Qualified Medicare Beneficiary program above 100% of the FPL.^30^ The Inflation Reduction Act further improved affordability by expanding full LIS benefits in 2024 to those with incomes between 135% and 150% of the FPL, eliminating Part D premiums and deductibles and introducing capped copayments.^31^

Finally, our study of the joint distribution of health care affordability challenges reveals that, while relatively few beneficiaries experienced all 3 issues—financial burden, financial barriers to care, and medical debt—a substantial proportion faced at least 1 or 2 affordability challenges. These are distinct yet interconnected concepts, each with different implications for beneficiaries. Financial barriers to care arise before treatment, often leading individuals to delay or forgo necessary medical services due to cost concerns. In contrast, financial burden and medical debt emerge after care has been received, reflecting difficulties in managing medical expenses. Although financial barriers primarily affect access to care, financial burden and medical debt are associated with ongoing economic strain, exacerbating financial insecurity and limiting individuals’ ability to seek future care. Understanding these distinctions is critical for informing policy interventions aimed at reducing health care affordability challenges.

### Limitations

Our study has some limitations. First, our sample was limited to the noninstitutionalized US population, excluding individuals in nursing homes and residential treatment facilities. Second, although we used validated measures to assess health care affordability problems, these measures relied on self-reported data, which may be subject to reporting error. Third, we could not control for all potential confounding factors; thus, our results should not be interpreted as evidence of a causal relationship. Fourth, our analysis used income as the primary criterion for determining eligibility for financial assistance programs. However, this approach has limitations, as we did not account for assets, which are also considered in eligibility determinations for certain subsidy programs. Fifth, there was inconsistency in the unit of analysis, as some outcomes were measured at the household level while others were assessed at the individual level. Sixth, we could not adjust for geographic factors such as rurality or state of residence, which may affect our findings given regional differences in health care costs and access.

## Conclusions

In this cross-sectional study of Medicare beneficiaries, beneficiaries with low income and beneficiaries with near low income had lower OOP health care spending compared with beneficiaries with middle or high income. However, Medicare beneficiaries with near low income—who qualify for only partial financial assistance with Medicare costs—experienced disproportionately greater financial hardship associated with health care expenses. Despite having a similar health status asbeneficiaries with low income, beneficiaries with near low income faced a critical coverage gap, lacking adequate financial protections while struggling to afford necessary care. These findings underscore the need for targeted policy interventions to address economic challenges among beneficiaries with near low income.

## Notes

Supplement 1. **eFigure 1.** Sample Selection Process **eFigure 2.** Trends in Health Care Utilization and Financial Affordability

Supplement 2.
Data Sharing Statement
