# Supplementary material for: Health Care Affordability Problems by Income Level and Subsidy Eligibility in Medicare
Source: JAMA Netw Open. 2025 Sep 22;8(9):e2532862. doi: 10.1001/jamanetworkopen.2025.32862 (PMC12455370; doi:10.1001/jamanetworkopen.2025.32862)
Supplement: Supplement 1. — eFigure 1. Sample Selection Process eFigure 2. Trends in Health Care Utilization and Financial Affordability [file jamanetwopen-e2532862-s001.pdf]

Supplementary Online Content

Park S, Fung V. Health care affordability problems by income level and subsidy eligibility in Medicare. *JAMA Netw Open.* 2025;8(9):e2532862.
doi:10.1001/jamanetworkopen.2025.32862

eFigure 1. Sample Selection Process
eFigure 2. Trends in Health Care Utilization and Financial Affordability

This supplementary material has been provided by the authors to give readers additional information about their work.

© 2025 Park S et al. *JAMA Network Open.*

eFigure 1. Sample Selection Process

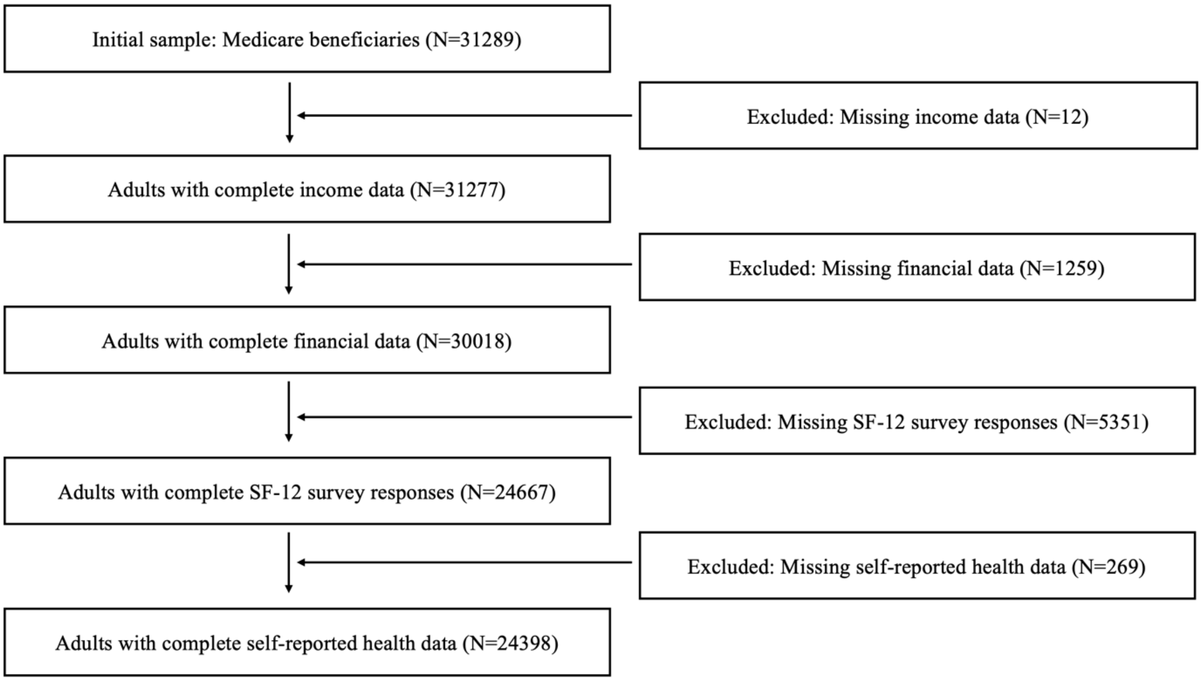

© 2025 Park S et al. *JAMA Network Open.*

eFigure 2. Trends in Health Care Utilization and Financial Affordability

Panel A. Health care utilization

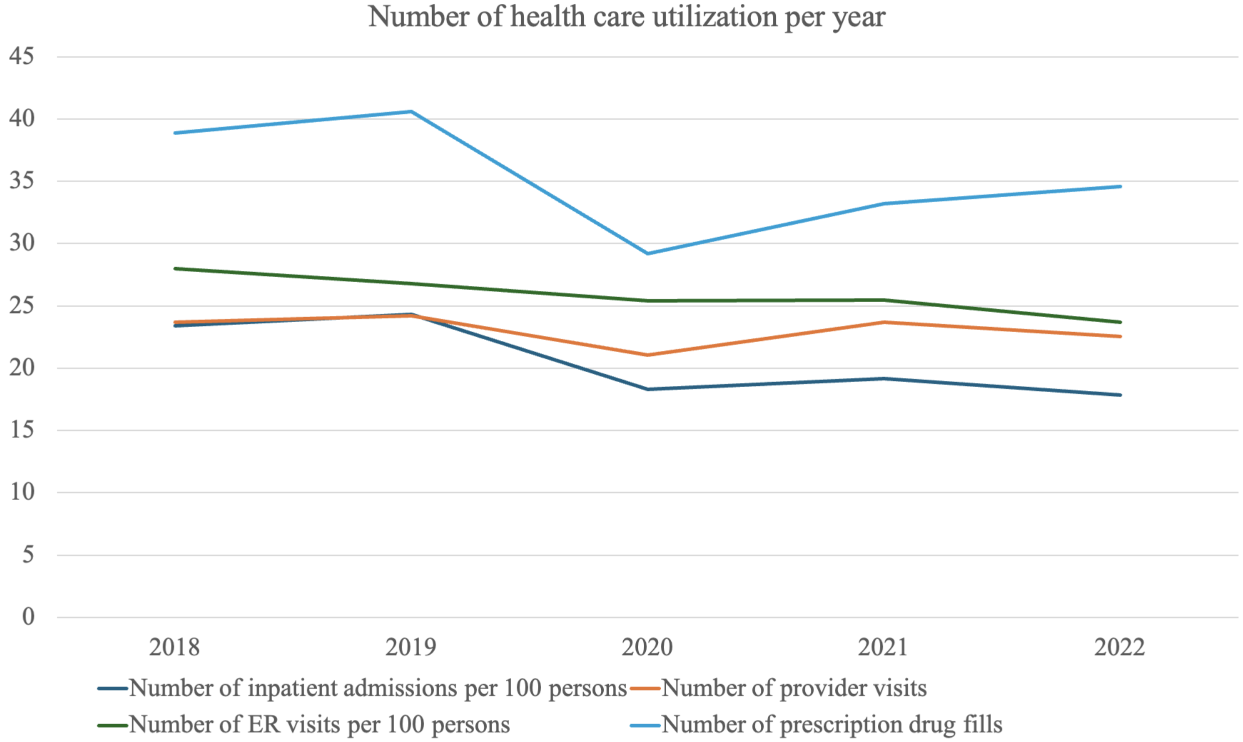

Panel B. Health care financial affordability

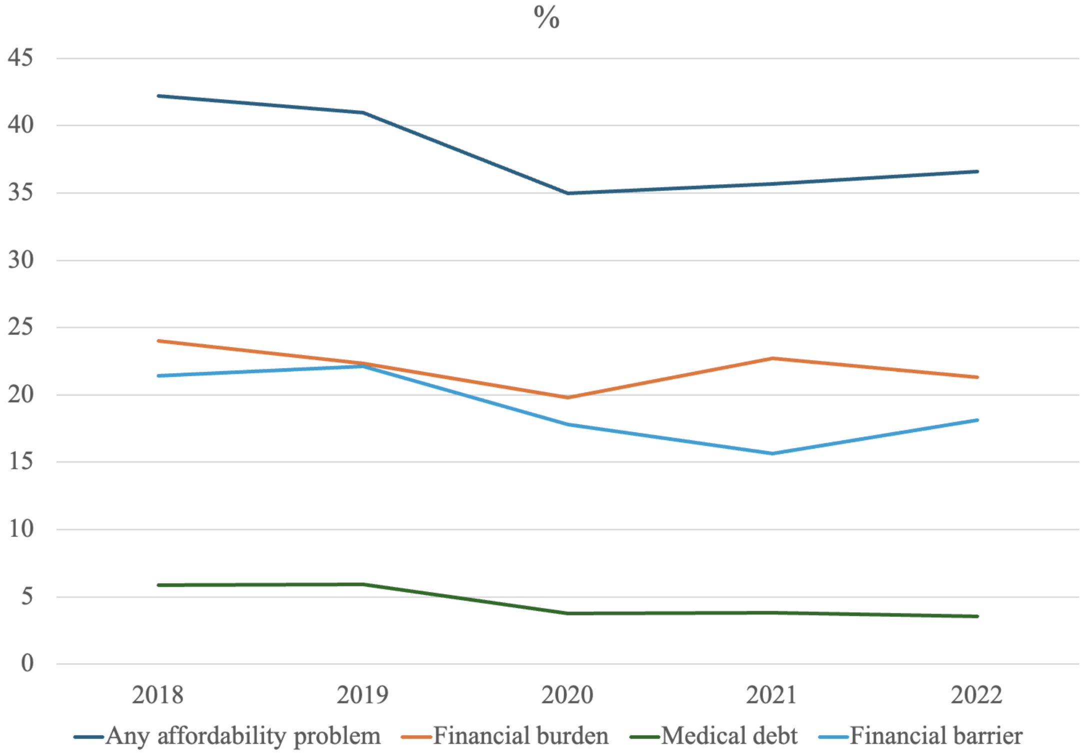

© 2025 Park S et al. *JAMA Network Open.*
