# Supplementary material for: Health Care Affordability Problems by Income Level and Subsidy Eligibility in Medicare
Source: JAMA Netw Open. 2025 Sep 22;8(9):e2532862. doi: 10.1001/jamanetworkopen.2025.32862 (PMC12455370; doi:10.1001/jamanetworkopen.2025.32862)
Supplement: Supplement 2. — Data Sharing Statement [file jamanetwopen-e2532862-s002.pdf]

Data Sharing Statement

Park. Health Care Affordability Problems by Income Level and Subsidy Eligibility in Medicare. *JAMA Netw Open*. Published September 22, 2025. doi:10.1001/jamanetworkopen.2025.32862

Data
Data available: No

Additional Information
Explanation for why data not available: The data is publicly available, but we're happy to share the dataset upon request
